# Supplementary figures and images for: Root application of Bisphenol A (BPA) and di(2-ethylhexyl) phthalate (DEHP) at environmental doses impacts tomato growth and production
Source: PLoS One. 2025 Oct 6;20(10):e0330476. doi: 10.1371/journal.pone.0330476 (PMC12500100; doi:10.1371/journal.pone.0330476)

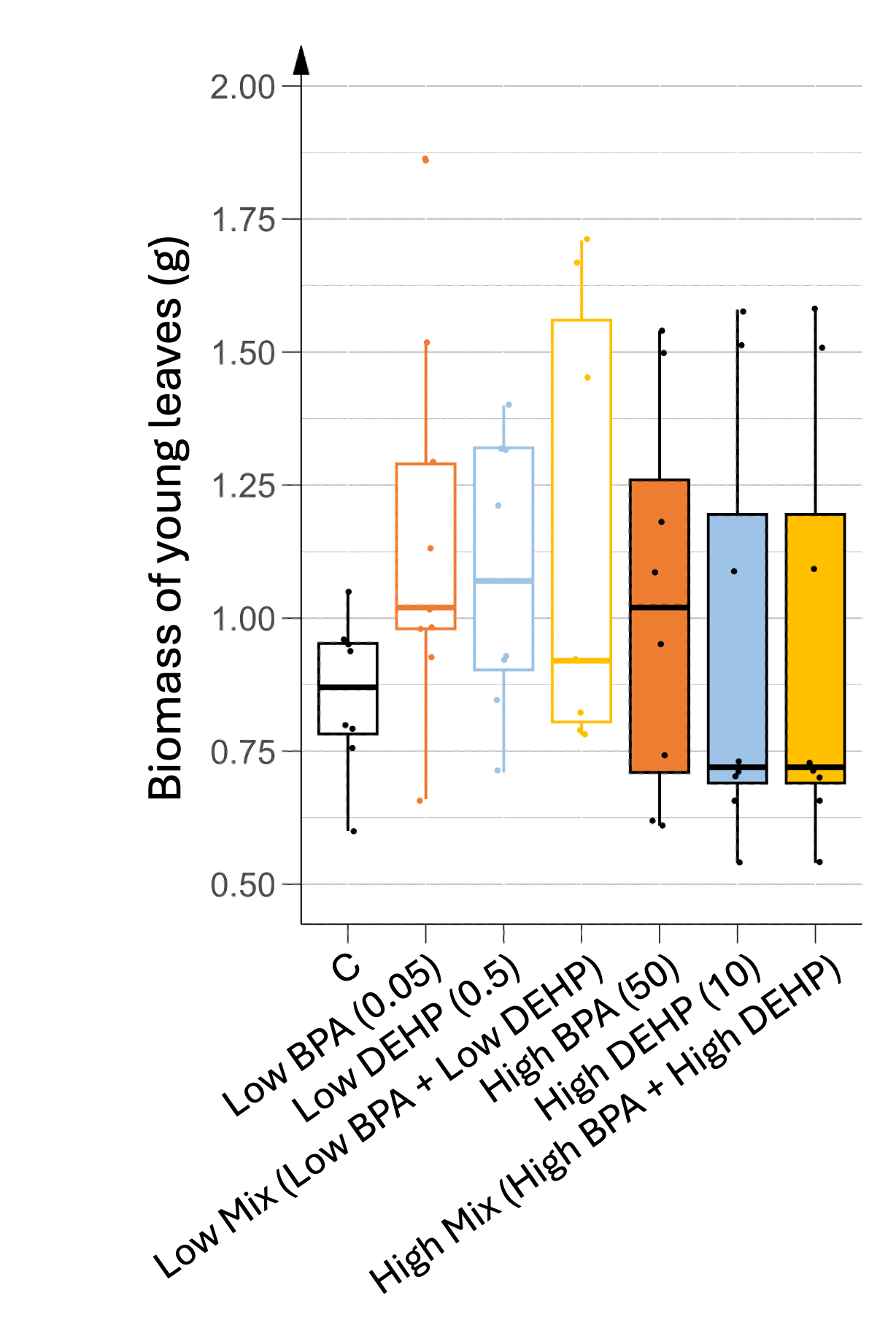

Supplement: S1 Fig — A pool containing leaves 6, 7, 8 and 9 were recorded. No significance was founded when Kruskal-Wallis test was used. (TIF) [file pone.0330476.s001.tif]

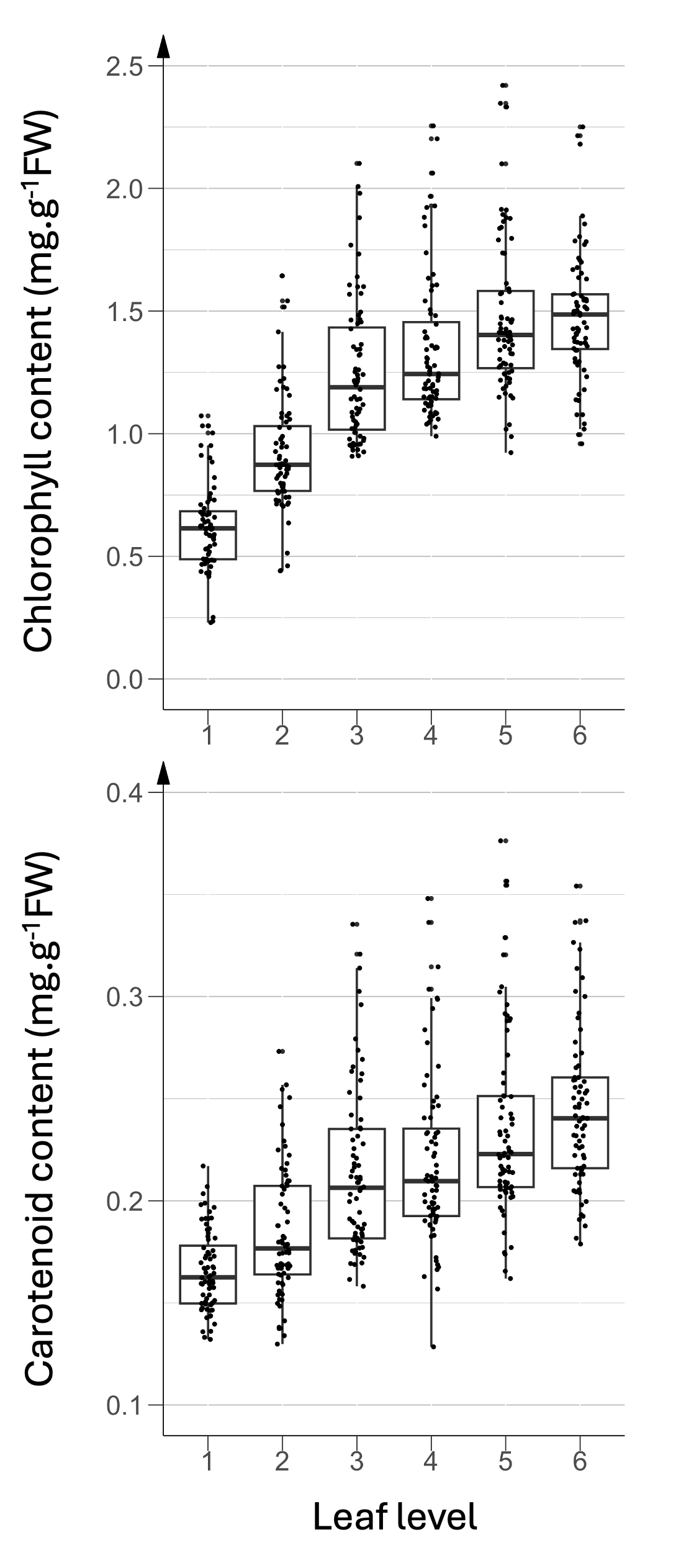

Supplement: S2 Fig — (A) chlorophyll a and (B) carotenoid contents in each leaf level ranging from the first leaf (1) produced to a pool of the last younger leaves (6) produced. (TIF) [file pone.0330476.s002.tif]

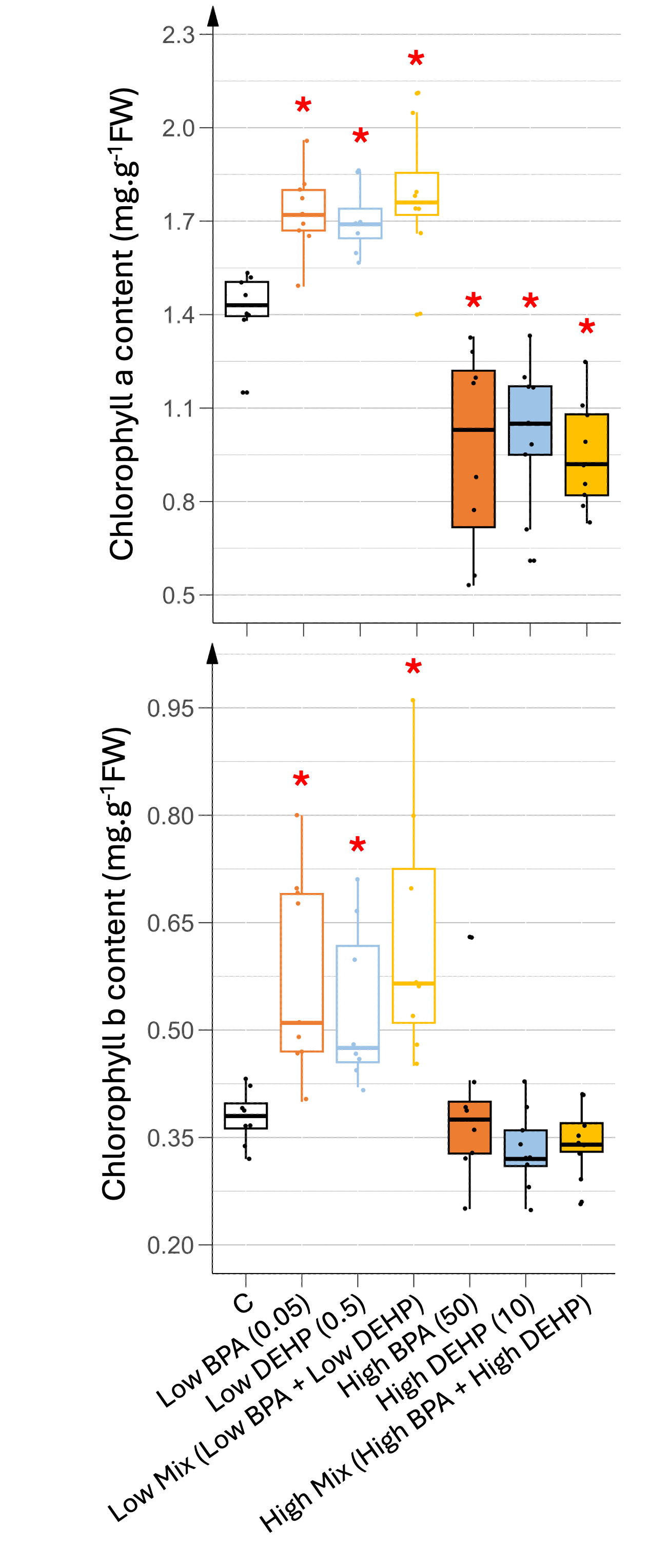

Supplement: S3 Fig — (A) chlorophyll a and (B) chlorophyll b. Stars represent significant differences between each EDC treatment against control using (a) Welch test and Dunnett’s test and (b) Kruskal-Wallis test and post hoc of Dunn; p-value < 0.05 (*); p-value < 0.1 (.). (TIF) [file pone.0330476.s003.tif]

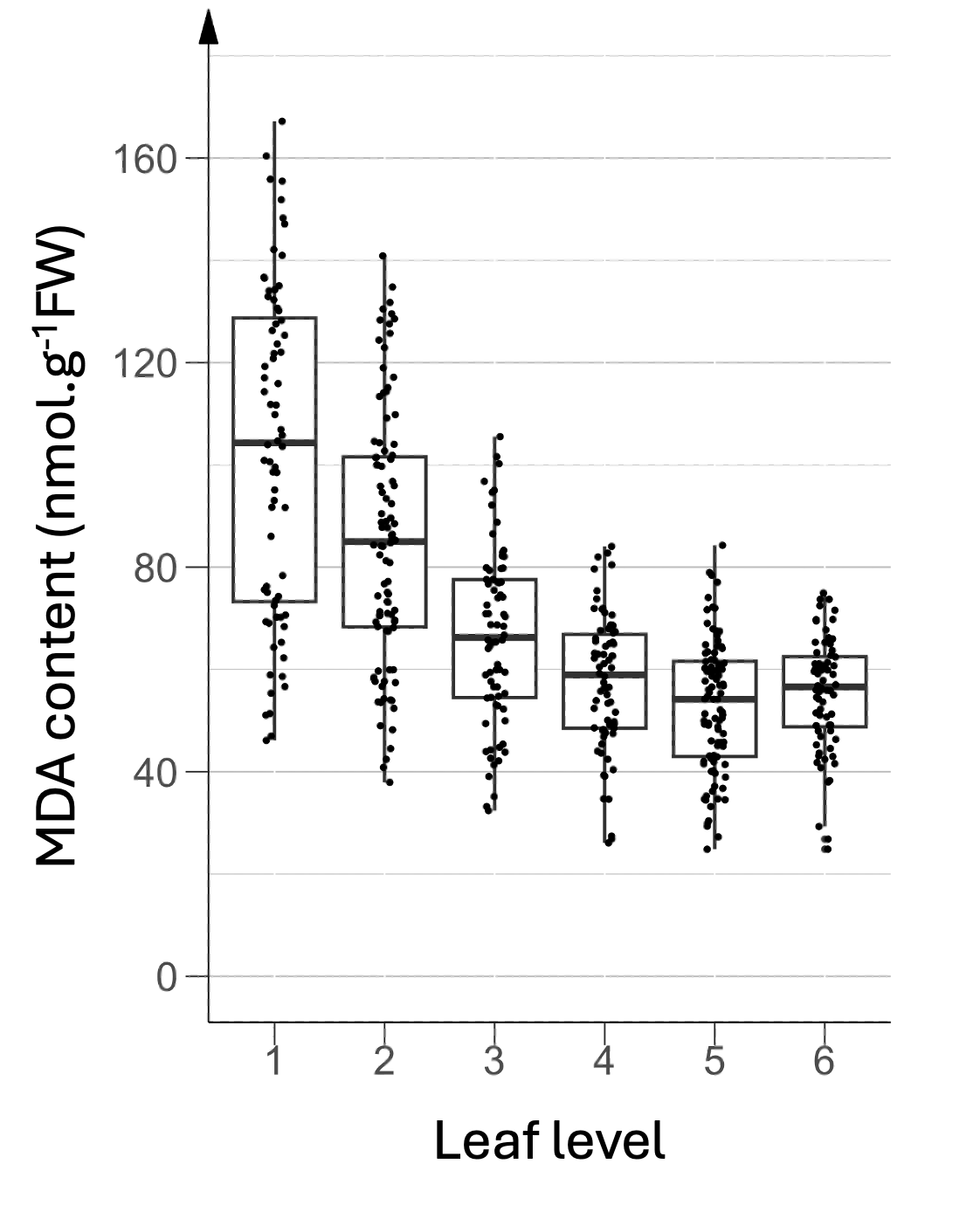

Supplement: S4 Fig — MDA contents were recorded in each leaf level ranging from the first leaf produced (1) to a pool of the last younger leaves (6) produced. Each level is a mix of all treatments. No significance was founded when Kruskal-Wallis test was used. (TIF) [file pone.0330476.s004.tif]

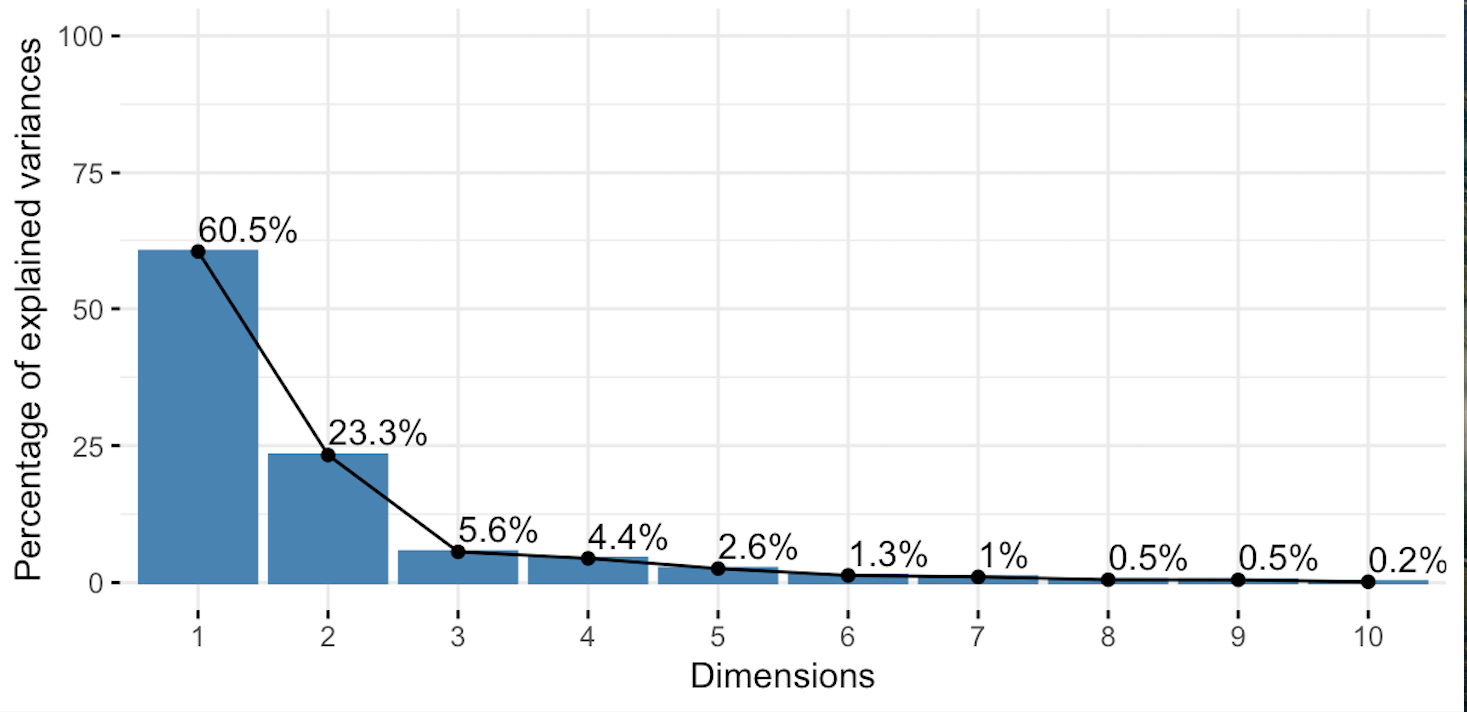

Supplement: S5 Fig — (TIF) [file pone.0330476.s005.tif]

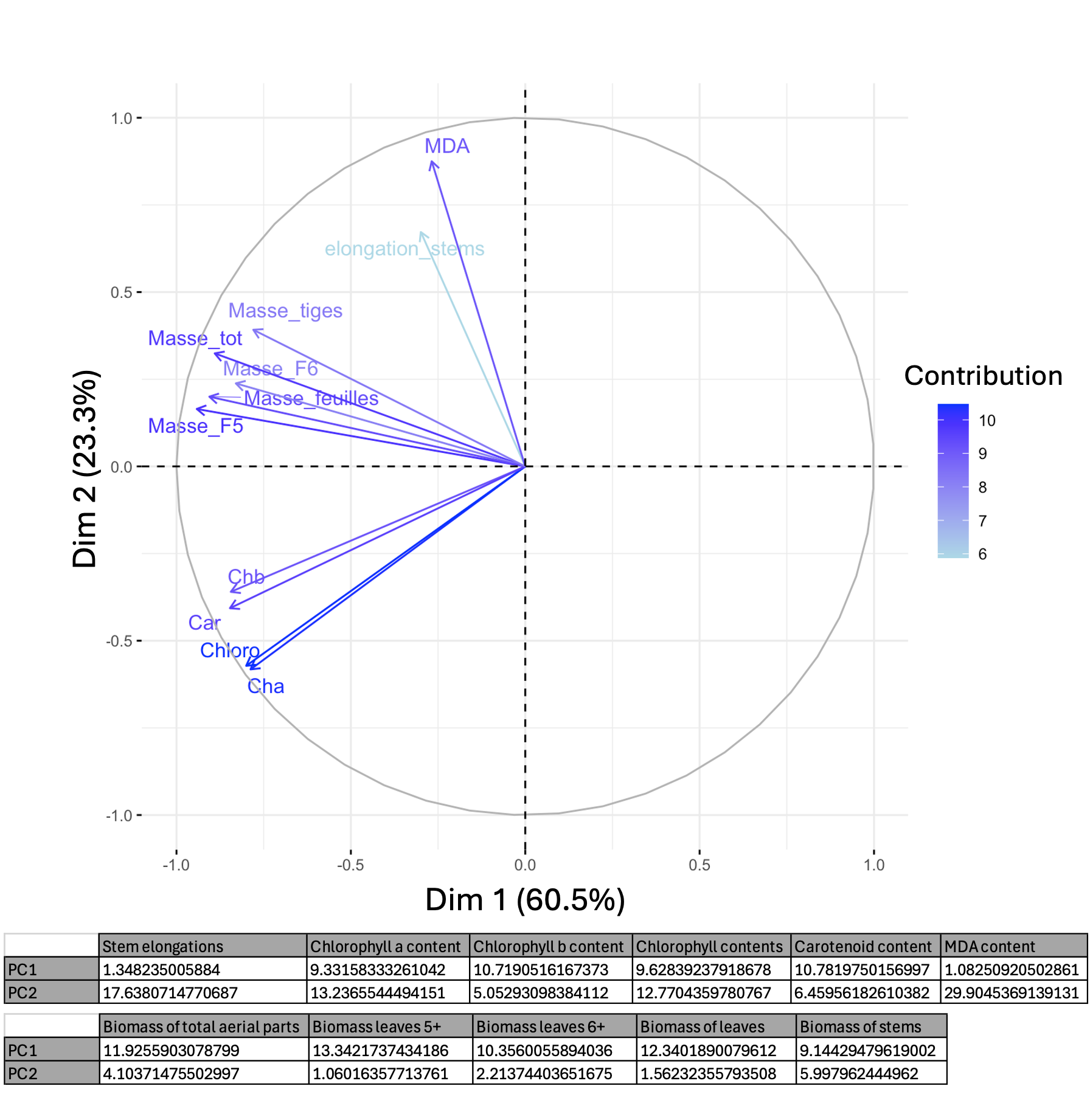

Supplement: S6 Fig — 11 morpho-physiological parameters during the vegetative phase are represented in the two PCs ranging from the most contributing one in dark blue to the less contributing one in light blue, with values inside the table below. (TIF) [file pone.0330476.s006.tif]

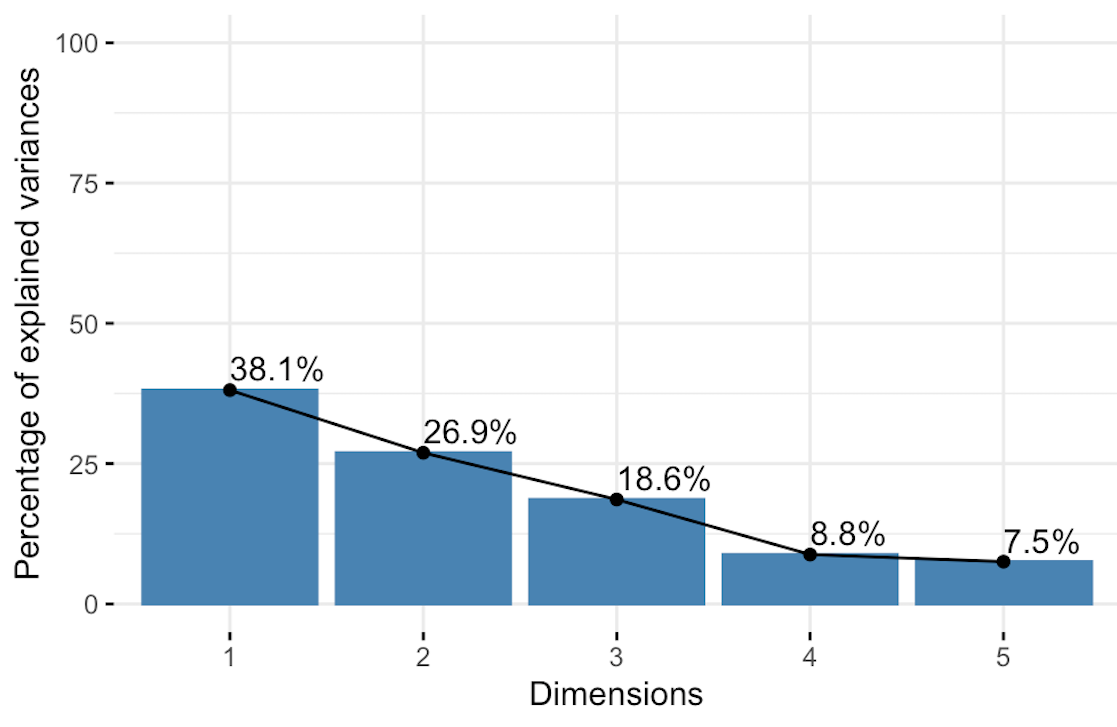

Supplement: S7 Fig — (TIF) [file pone.0330476.s007.tif]

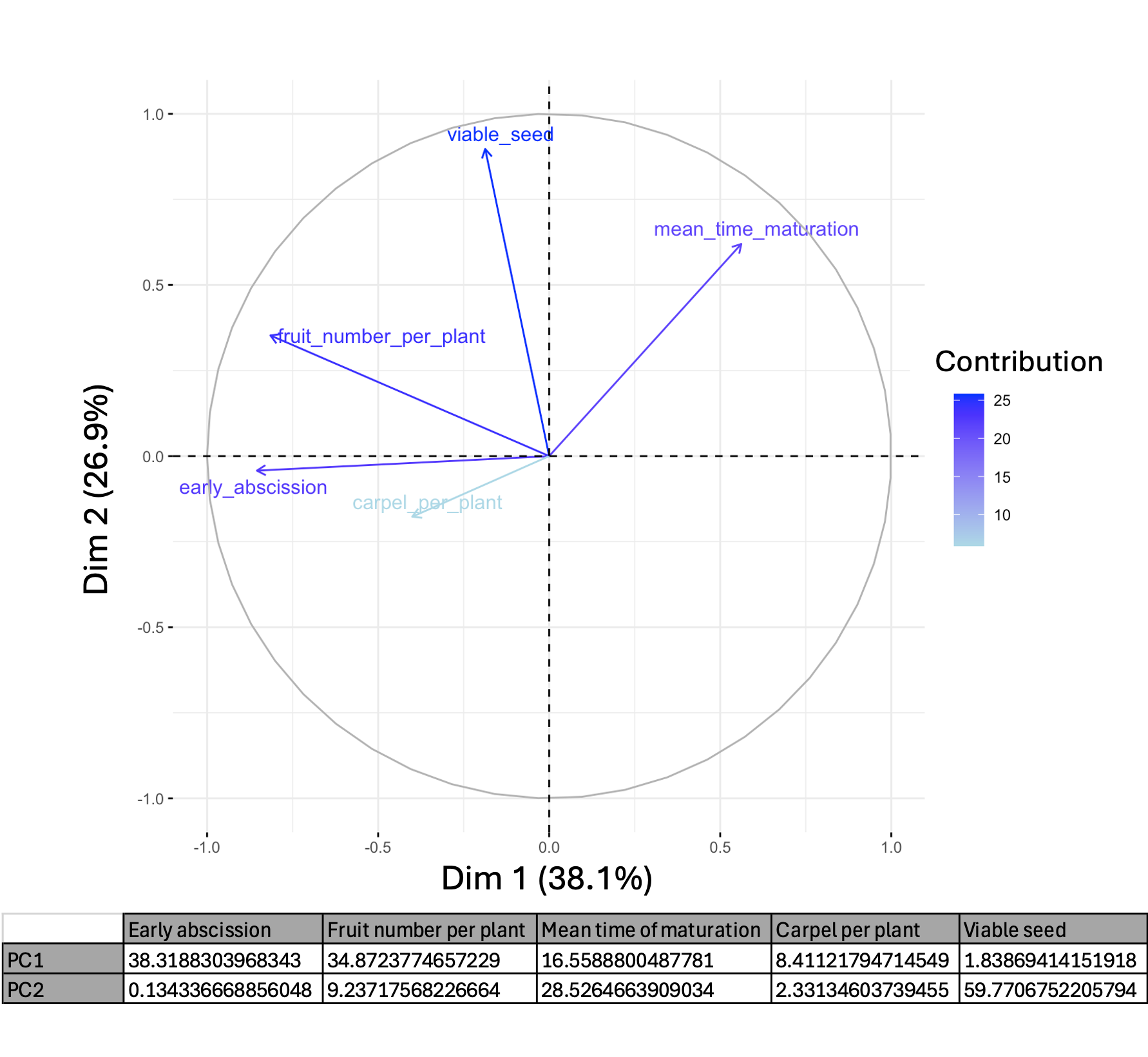

Supplement: S8 Fig — 5 morpho-physiological parameters during the reproductive phase are represented in the two PCs ranging from the most contributing one in dark blue to the less contributing one in light blue, with values inside the table below. (TIF) [file pone.0330476.s008.tif]
